# Complete Genome Sequence of Avian Influenza Virus A/chicken/Jiangsu/1001/2013(H5N2), Demonstrating Continuous Reassortance of H5N2 in China

**DOI:** 10.1128/genomeA.00469-13

**Published:** 2013-07-18

**Authors:** Zhiqiang Mi, Wei Liu, Hang Fan, Xiaoping An, Guangqian Pei, Wei Wang, Xiaomeng Xu, Maijuan Ma, Zhiyi Zhang, Wuchun Cao, Yigang Tong

**Affiliations:** State Key Laboratory of Pathogen and Biosecurity, Beijing Institute of Microbiology and Epidemiology, Beijing, China

## Abstract

Avian influenza virus A/chicken/Jiangsu/1001/2013(H5N2) was identified from a healthy chicken in an eastern China poultry market. Whole-genome analysis demonstrated that the H5N2 virus originated from a reassortance between a previous A/chicken/Hebei/1102/2010(H5N2) virus and an endemic H5N1 virus. The results indicated that continuing reassortance of H5N2 has been occurring in domestic poultry of China.

## GENOME ANNOUNCEMENT

From 2000 onwards, various reassortant viruses with the hemagglutinin (HA) gene from the influenza virus A/goose/Guangdong/1/96-like lineage and internal segments from other subtypes emerged in chickens, ducks, and geese (1–3). In Asia, the H5N2 virus was found mainly in migratory birds, and recently it was isolated from poultry and mammals. In 2010, four novel reassortant H5N2 avian influenza viruses were isolated from poultry in China, and the characterization demonstrated that these novel reassortant H5N2 viruses could not be grouped in the HA tree with those H5N2 viruses reported previously in Asia and were derived from reassortance between circulating highly pathogenic avian influenza (HPAI) H5N1 and endemic H9N2 viruses (3).

In this study, 20 feces samples from apparently healthy chickens in a live bird market in the Jiangsu province of eastern China were collected and used to screen for avian influenza virus infection with the primers and conditions described by Zhou et al. (4). The obtained fragments were subjected to high-throughput sequencing, and a complete genome sequence of the avian influenza virus was assembled. A feces sample from a chicken resulted in a positive amplification, and the sequencing results showed that the chicken was infected with an H5N2 avian influenza virus, named A/chicken/Jiangsu/1001/2013(H5N2) (CK/JS/1001/13). No other subtypes of avian influenza virus (AIV) were detected in the same feces sample. Genomic analysis showed that seven segments of CK/JS/1001/13 had the highest homology to corresponding gene segments from A/chicken/Hebei/1102/2010(H5N2) (CK/HB/1102/10), which was isolated in 2010 in a live bird market in northern China and confirmed to have originated from a reassortance between a clade 7 H5N1 virus and an endemic H9N2 virus (3). The matrix protein gene of CK/JS/1001/13 was found to derive from a clade 7.2 endemic H5N1 virus but not H9N2-like CK/HB/1102/10. The result suggested that the recently identified CK/JS/1001/13 virus originated from a reassortance between CK/HB/1102/10(H5N2) and an endemic H5N1 virus.

The long-term endemicity of the highly pathogenic avian influenza H5N1 virus in poultry and the common practice of intermingling in poultry raising were thought to be possible reasons for the generation of reassortant H5 HPAI viruses with neuraminidase (NA) subtypes other than N1 (3). Although no other subtypes of avian influenza virus were identified in the 20 feces samples, this study also supports the idea that H5N1 functions as a basis for reassortance and that reassortant events have been occurring in poultry in China.

### Nucleotide sequence accession numbers.

The complete genome sequence of A/chicken/Jiangsu/1001/2013(H5N2) is available in GenBank under accession numbers KF150631, KF150632, KF150633, KF150634, KF150635, KF150636, KF150637, and KF150638.
